# China Public Psychology Analysis About COVID-19 Under Considering Sina Weibo Data

**DOI:** 10.3389/fpsyg.2021.713597

**Published:** 2021-09-08

**Authors:** Wei Pan, Ren-jie Wang, Wan-qiang Dai, Ge Huang, Cheng Hu, Wu-lin Pan, Shu-jie Liao

**Affiliations:** ^1^School of Applied Economics, Renmin University of China, Beijing, China; ^2^Department of Obstetrics and Gynecology, Tongji Medical College, Tongji Hospital, Huazhong University of Science and Technology, Wuhan, China; ^3^School of Economic and Management, Wuhan University, Wuhan, China

**Keywords:** public psychology, COVID-19, Sina Weibo data, China, sentiment classification model

## Abstract

COVID-19 not only poses a huge threat to public health, but also affects people’s mental health. Take scientific and effective psychological crisis intervention to prevent large-scale negative emotional contagion is an important task for epidemic prevention and control. This paper established a sentiment classification model to make sentiment annotation (positive and negative) about the 105,536 epidemic comments in 86 days on the official Weibo of People’s Daily, the test results showed that the accuracy of the model reached 88%, and the AUC value was greater than 0.9. Based on the marked data set, we explored the potential law between the changes in Internet public opinion and epidemic situation in China. First of all, we found that most of the Weibo users showed positive emotions, and the negative emotions were mainly caused by the fear and concern about the epidemic itself and the doubts about the work of the government. Secondly, there is a strong correlation between the changes of epidemic situation and people’s emotion. Also, we divided the epidemic into three period. The proportion of people’s negative emotions showed a similar trend with the number of newly confirmed cases in the growth and decay period, and the extinction period. In addition, we also found that women have more positive emotional performance than men, and the high-impact groups is also more positive than the low-impact groups. We hope that these conclusions can help China and other countries experiencing severe epidemics to guide publics respond.

## Introduction

A novel coronavirus was first reported in Wuhan, Hubei in early December 2019 (Li Q. et al., 2020; Zhu et al., 2020), followed by a series of outbreaks around the world in the following months. On February 11, 2020, WHO announced the official designation of the virus as “COVID-19.” In response to the COVID -19 epidemic, the Chinese government has adopted a series of active and effective strategies, and achieved the basic control of the epidemic by March 2020. However, the epidemic situation outside of China is still severe. As of May 21, 2020, the cumulative number of infections in the world has exceeded 5 million and the number of deaths has exceeded 300,000. the cumulative number of infections in the world has exceeded 5 million and the number of deaths has exceeded 300,000. This public health emergency not only jeopardizes people’s physical health, but also affects people’s mental health (Holmes et al., 2020; Moccia et al., 2020; Wang et al., 2020). On January 26, the National Health Commission of China issued the guiding principles for COVID-19 emergency psychological crisis intervention (Liu et al., 2020), incorporating psychological crisis intervention into the overall deployment of epidemic prevention and control, so as to reduce psychological harm caused by the epidemic and promote social stability. Some scholars have also analyzed the negative emotions revealed by people during the epidemic, such as fear, sadness, anxiety, depression, etc. (Bitan et al., 2020; Brooks et al., 2020; McKay et al., 2020; Wallace et al., 2020). Most of these studies use questionnaires and scales to analyze the mechanism of people’s negative emotions.

However, how widespread are negative emotions? How do people’s emotions change with the development of the epidemic? Such problems have not yet been studied. It cannot be ignored that emotion can be transmitted just like any other type of information, which is called emotional contagion (Barsade, 2002; Del Vicario et al., 2016; Zhang G. et al., 2018), and this phenomenon is especially obvious in social networks (Kramer et al., 2014). Therefore, exploring the changing laws of online public opinion has a positive effect on preventing the spread of negative emotions. After the outbreak of COVID-19, the growing social network platform has become the main place for people to pay attention to the epidemic and express their emotional appeals. In China, Sina Weibo is the most representative online social media (Li and Xu, 2014; Yuan and Gao, 2016), through which people can know the real-time dynamics of the epidemic and freely comment on the news and topics. These comments contain the subjective feelings of the reviewers, which can be divided into two different dimensions of positive and negative in psychology (Cacioppo et al., 1999; Watson et al., 1999). Understanding the evolution of public emotion is helpful for the government to effectively respond to online public opinions (Li S. et al., 2020). China is the country with the earliest outbreak of COVID-19, and it has gone through a complete process from the beginning, outbreak to control. We hope that by analyzing the evolution of online public opinion of COVID-19 in China, we can provide some reference and help to other countries experiencing severe epidemic to prevent large-scale negative emotional contagion and ensure people’s mental health.

The recognition and division of comment information is the premise of analyzing the law of public emotion evolution. Therefore, we have to mention the concept of text sentiment analysis, which is a process of analyzing, processing and extracting subjective text with emotional color by using natural language processing and text mining technology (Bakshi et al., 2016). At present, the research on text sentiment analysis is mainly divided into semantic-based emotion dictionary method (Al-Thubaity et al., 2018; Jha et al., 2018; Zhang S. et al., 2018; Xing et al., 2019) and machine learning method (Catal and Nangir, 2017; Abdi et al., 2019; Zhang et al., 2019; Xu F. et al., 2020). The former is mainly based on the existing emotion dictionary or semantic database to weighted sum the words with emotion or polarity in the text, while the latter is mainly to build feature vectors with category representation meaning in the text, and then use machine learning algorithm to classify based on these feature vectors. Some scholars believe that the semantic-based emotion dictionary method should be combined with the specific context, and its effectiveness needs to be further verified (Kumar et al., 2020). Therefore, the machine learning method is more flexible and applicable.

The main work of this paper is to explore the emotional evolution of Weibo users during the epidemic in China through text sentiment analysis. First of all, through the technology of web crawler, users’ comments on the epidemic in the People’s Daily official Weibo from December 31, 2019 to March 31, 2020 were obtained. Secondly, part of the data was randomly selected for artificial sentiment annotation, and the marked data set was divided into training set and test set. Thirdly, based on the SnowNLP module in Python, the naive Bayesian algorithm in machine learning was used to build sentiment classification model, and the automatic recognition and division of the remaining comment information was completed. Finally, the annotated data set was used to explore the potential law between the changes of emotion and epidemic situation.

## Materials and Methods

### Data Acquisition

**FIGURE 1:**
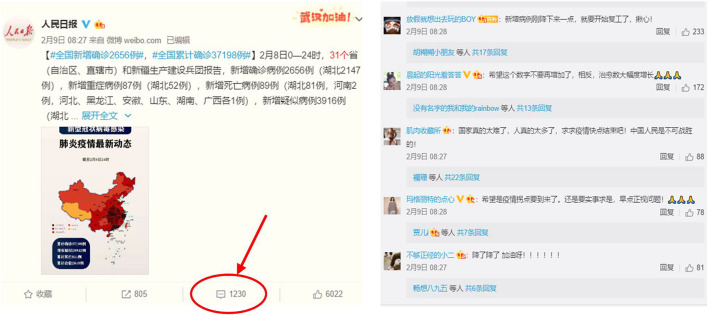
Weibo user comments.

Our data comes from the comments on the daily epidemic data released on the official Weibo of People’s Daily from December 31, 2019 to March 31, 2020, as shown in the example in Figure 1. During this time period, China’s COVID-19 epidemic experienced the entire process from outbreak to being controlled. People’s Daily is the most authoritative and influential official media in China, and its Weibo has a high degree of concern. Moreover, it is timely and continuous for the update of the epidemic development, and people often express their emotional appeals and opinions on their weibo. Therefore, this paper chooses the People’s Daily as the carrier to obtain comments. The requests library in python is used to simulate logging into Sina Weibo for web crawler to obtain data. The information crawled includes comment date, comment content, reviewer gender, number of fans, etc. In the early stage of the epidemic in China, no data was released for some certain dates (2020/01/01, 2020/01/04, 2020/01/07, 2020/01/09, 2020/01/010, 2020/01/14). After deleting these dates, 112588 original comments within 86 days were crawled and stored in an Excel table.

### Data Preprocessing

Text data is an unstructured data. Due to different habits, people usually use a variety of complex emoji, symbols and expressions in their comments, which greatly increase the difficulty of text recognition. Data preprocessing is the key to improving the accuracy of text recognition. Its processing method is different from that of structured data. In addition to the processing of missing and repeated values, it also includes the cleaning of text content.

#### Missing Value and Duplicate Value Processing

Since the data in this paper is obtained by web crawlers, there are almost no missing values in the data set. But some comments contain only an emoji, symbol, or character, which are invalid information and are deleted as missing values. We also eliminated duplicate values in the data set to ensure the uniqueness of the data.

#### Text Content Cleaning

The comment text contains some noise information unrelated to the study, such as URL, punctuation, symbol, forwarding, picture, etc., which cannot reflect the user’s emotional inclination and will interfere with the recognition of the text. Therefore, we use regular expressions to locate and delete this information, ensuring the consistency and readability of all comments in the format.

After data preprocessing, this data set covers 105,536 comments on epidemic data released by People’s Daily, which are stored in chronological order, including comment date, comment content, number of fans and commentator gender, as shown in Table 1.

**TABLE 1:** Comments after preprocessing.

**Date**	**Content**	**Fans**	**Gender**
2019/12/31	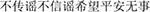	2,069,206	Female
	(Do not spread rumors, do not believe the rumors, hope it is safe)		
2019/12/31	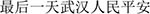	108	Female
	(Hope people in Wuhan are safe on the last day of 2019)		
2019/12/31	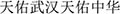	84	Male
	(God bless Wuhan, God bless China!)		
2019/12/31	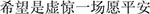	548,370	Female
	(Hope this is a false alarm!)		
2019/12/31	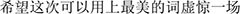	167	Female
	(Hope I could use the most beautiful word this time: false alarm)		
……	……	……	……

### Artificial Sentiment Annotation and Data Set Division

For the pre-processed data set, we randomly selected part of the data for artificial sentiment annotation, and divided the reviewer’s comments into three categories: Positive, negative and neutral. However, during the labeling process, we found that most of the comments focused on the positive and negative categories, and there are very few comments actually classified as neutral emotions (less than 5%). The small sample size makes it difficult for the machine learning model to identify neutral emotions well, and it also affects the overall fitting effect of the model. Therefore, after comprehensive consideration, we only divided the comments into two categories: positive (non-negative, including neutral) and negative. The specific annotation rules are shown in Table 2. We randomly marked 6,500 comments, of which 5,000 were used as training sample set (2,500 positive and 2,500 negative emotions) to construct the model, and 1,500 were used as the test set to verify the accuracy of the model. Some of the annotated comments are shown in Table 3.

**TABLE 2:** Emotion classification principles.

**Sentiment classification**	**Definition**	**Label**
Positive emotions	Emotions arising from an increase in positive value or a decrease in negative value, such as pleasure, trust, gratitude, rejoicing, expectation, praise, etc.	1
Negative emotions	Emotions arising from a decrease in positive value or an increase in negative value, such as pain, anxiety, anger, frustration, hatred, jealousy, irony, doubt, etc.	–1

**TABLE 3:** Artificial sentiment annotation comments.

**Date**	**Content**	**Label**
2019/12/31	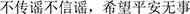	1
	(Do not spread rumors, do not believe the rumors, hope it is safe.)	
2020/3/12	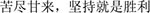	1
	(Perseverance is victory.)	
2020/2/20	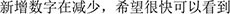	1
	(New confirmed cases are decreasing.)	
2020/2/9	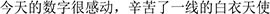	1
	(Today’s numbers are very moving. Thanks for the angels in white!)	
2020/1/25	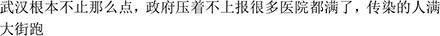	–1
	(Wuhan is more than that. The government has suppressed the report, many hospitals are full and infected people are all over the street)	
2020/2/4	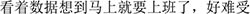	–1
	(Looking at the data and thinking about going to work soon, so terrible)	
2020/2/2	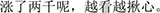	–1
	(It’s up 2,000 cases. I’m really worried)	
2020/3/29	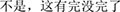	–1
	(There’s no end to it.)	
……	……	……

### SnowNLP Sentiment Analysis

SnowNLP is a class library developed by Python, which is mainly used to process Chinese text content. Its main functions include word segmentation, part-of-speech tagging, sentiment analysis, keyword extraction, etc. This paper mainly uses the sentiment analysis function to construct a sentiment classification model based on the marked 6,500 comments to complete the marking of the remaining comments. The typical steps of this kind of labeling task include word segmentation processing for comments, then removing stop words, and finally using a polynomial Bayes classifier to classify emotions. The specific steps are as follows.

**Step 1: Word Segmentation**

Chinese word segmentation technology refers to dividing a sequence of Chinese characters into individual words according to their semantics. Existing word segmentation algorithms can be divided into three categories: based on string matching, based on understanding and based on statistics. The word segmentation of the SnowNLP library is implemented through the Character-Based Generative Model (Wang et al., 2009), which is one of the statistical word segmentation methods. The model adopts the maximum joint probability to model the optimal word segmentation scheme. For a sentence $c_{1}^{n}$ with *n* characters, the optimal word segmentation $W\,S\,e\,q=w_{1}^{m}=w_{1},w_{2}\,\cdots ,w_{m}$ shall meet the following requirements:

$$W\,S\,e\,q^{*}=\arg\,\max_{W\,S\,e\,q}P\,\left(w\,s\,e\,q|c_{1}^{n}\right)$$

Word segmentation can also be used to count the frequency of vocabulary appearing in the text and generate a visual word cloud image, so as to analyze the common concerns and psychological characteristics of Weibo users during the epidemic.

**Step 2: Remove stop words**

Stop words refer to the words that appear frequently in the text but have little practical significance. It mainly includes modal particles, adverbs, prepositions and conjunctions. They usually have no specific meaning of their own, and are useful only when put into a complete sentence, such as “of,” “in,” “and” and,” “then,” etc. These words not only cannot be used as the recognition feature of text classification, but also cause some interference. Therefore, it is necessary to manually define the stop word list to delete the stop words in the word vector after word segmentation.

**Step 3: Naive Bayes classification**

The basic model of SnowNLP sentiment classification is the Naive Bayes model, which is one of the most commonly used text classification models. Word vectors can be obtained by word segmentation, and the category of the text is related to the words and frequencies appearing in the text. The text is represented by a set of feature vectors *T*(*t*_1_,*t*_2_,⋯,*t*_*n*_) that can distinguish its category. For a binary classification problem with two categories *c*_1_ and *c*_2_, to calculate which category the eigenvector *T* belongs to, the conditional probability *P*(*c*_*i*_|*T*) of each category should be calculated:

$$P\,\left(c_{i}|T\right)=\frac{P\,\left(T|c_{i}\right)\cdot P\,\left(c_{i}\right)}{P\,\left(T\right)}$$

Where:

$$P\,\left(T\right)=P\,\left(T|c_{1}\right)\cdot P\,\left(c_{1}\right)+P\,\left(T|c_{2}\right)\cdot P\,\left(c_{2}\right)$$

SnowNLP can divide sentiment by setting a probability threshold. The text whose conditional probability calculated by a certain category exceeds a given threshold is divided into this category, and those whose probability is less than the threshold will be divided into another category.

### Evaluation of the Model

To ensure that the sentiment classification model has good performance, we use the following metrics: accuracy (Acc), area under the ROC curve (AUC).

**Accuracy:** It can be expressed as the proportion of the correctly classified samples to the total sample, which is the most representative evaluation method. The calculation is as follows:

$$A\,c\,c\,u\,r\,a\,c\,y=\frac{T\,P+T\,N}{T\,P+F\,N+T\,N+F\,P}$$

**AUC:** The area under a receiver operating characteristic (ROC) curve. The *x*-axis of the ROC curve is the false positive rate and the *y*-axis is the true positive rate. In a proportionally unbalanced data set, this is a more comprehensive measure than accuracy.

### Evaluation of the Model

In this paper, we also use analysis of variance to test the significant differences between Weibo users after grouping. The basic idea of the analysis of variance is to divide the total variation (i.e., total variance) of the measurement data into processing (between groups) effects and error (within groups) effects according to the sources of variation, and then to determine the main source of differences through comparison.

The main steps of analysis of variance are:

(1) Decomposition of the total sum of squares
(2) Decomposition of total degrees of freedom
(3) F inspection.

If the *F*-test is significant, reject the null hypothesis that there is no significant difference.

## Results

### SnowNLP Data Annotation Results

**FIGURE 2:**
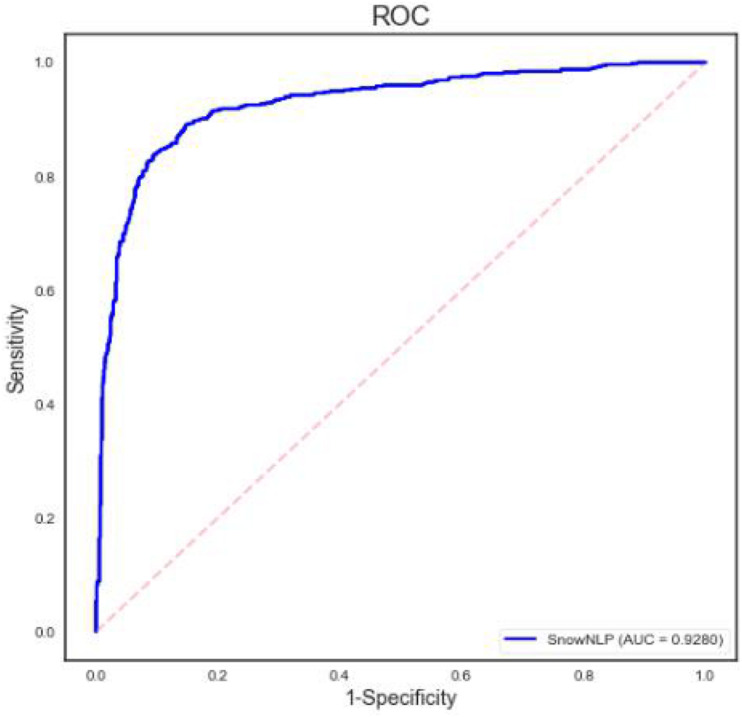
ROC curve.

Based on the SnowNLP module in python and the 6,500 comments manually annotated, we have completed the annotation of the remaining samples. In the process of model parameter debugging, we found that the model reach best fitting effect when the probability threshold of positive emotion was set to 0.7, and the fitting results of the model verified by 1,500 comments of the test set are shown in Table 4. It can be seen that the accuracy of the model has reached to 88%. The ROC curve in Figure 2 also shows that the value of AUC exceeds 0.9, proving that the training model has good generalization ability and can be used to predict the remaining sample labels. After SnowNLP completed the prediction, we got 105,536 comments with “positive” and “negative” tags, including 59,293 positive comments and 46,243 negative comments. Our subsequent research is based on the analysis of this data set.

**TABLE 4:** Fitting results.

	**Precision**	**Recall**	**F1-score**	**Support**
–1	0.89	0.92	0.90	922
1	0.86	0.82	0.84	578
Accuracy	0.88			1,500

### Public Opinion Focus and Sentiment Expression

**FIGURE 3:**
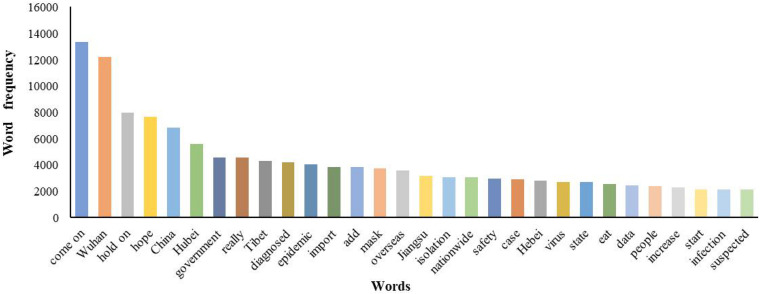
Word frequency statistics.

Figure 3 depicts the 30 words with the highest frequency in Weibo users’ comments. The words “come on” and “Wuhan” appear much more frequently than other words. “Wuhan” reflects the focus and discussion center of the epidemic in China, while “come on” indicates that people generally show positive emotions of support and encouragement in the face of the epidemic. In addition, words like “hold on,” “hope,” “safety” also express the people’s good wishes and expectations for the epidemic. The geographical terms “Wuhan,” “Hubei,” “Tibet,” “Jiangsu,” and “Hebei” express their concern about the spatial spread of the epidemic. The words “confirmed,” “add,” “data,” and “increase” express their concern about the changes in the number of COVID-19 cases. The words “China,” “government” and “state” reflect the public’s concern about the government’s response to the epidemic. The words “mask,” “isolation,” and “eat” reflect people’s concern about life security in the epidemic.

**FIGURE 4:**
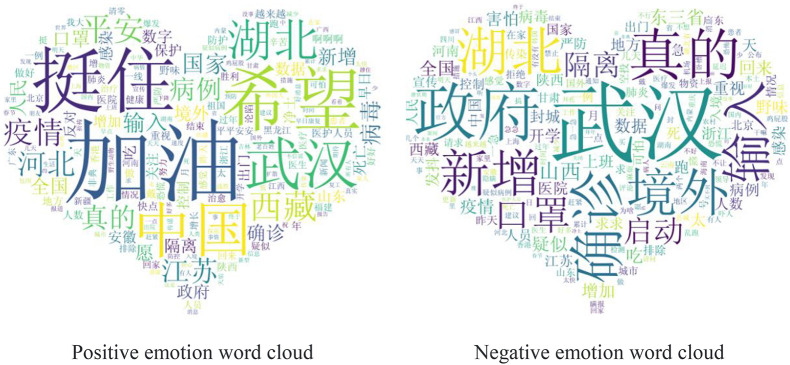
Word cloud of positive and negative comments.

Figure 4 shows the word cloud image of comment information marked as positive and negative emotions. The larger the font, the higher the word frequency. It can be seen that the words “come on,” “hold” and “hope” are more prominent in the word cloud of positive emotions, while the words “Wuhan,” “government” and “really” in the word cloud of negative emotions are more frequent. As Wuhan was the first city to find covid-19 cases in China, and the epidemic situation was the most severe, the negative emotions of the people were mostly related to it. The high frequency of the word “government” in negative emotions indicates that some people still doubts the government’s work. “Really” is mostly used to emphasize people’s negative emotions and the doubts about the epidemic data, such as “it’s really scary,” “it’s really hard to see the rising figures” and “is the data true.”

In general, according to the statistics of high-frequency words, the positive emotions of Weibo users are the majority during the epidemic, but it is undeniable that there are still some negative emotions, which mainly come from the epidemic itself and the work of the government.

### Epidemic Development and Public Sentiment Change

Statistics data of the epidemic is a direct way for the public to understand the development of the epidemic. The epidemic data released by the People’s Daily includes the newly added and cumulative “confirmed cases,” “cured cases,” “deaths,” “suspected patients,” “critically ill patients,” etc. Among them, the number of newly confirmed cases is one of the most concerned data indicators, which directly reflects the severity and development trend of the epidemic. According to the changes in the number of newly confirmed cases, the epidemic in China can be divided into three stages: Stage I (epidemic began—2020/02/04), the number of newly confirmed cases in this stage shows a clear upward trend, and we define it as the growing period of the epidemic; Stage II (2020/02/05—2020/03/05), the number of newly confirmed cases in this stage shows a significant downward trend. We define it as the decay period of the epidemic. Among them, the reason for the sharp increase on the day of 2020/02/12 is that the statistical caliber has changed. The statistical agencies included the number of clinically diagnosed cases into the number of confirmed cases, and the sharp change in the value does not represent the inflection point of the epidemic; Stage III (after 2020/03/05), the characteristic of this stage is that the number of newly confirmed cases per day tends to be stable, and no more than 100 newly confirmed cases a day, the proportion of new cases imported from abroad gradually increases. We define it as the extinction period of the epidemic.

**FIGURE 5:**
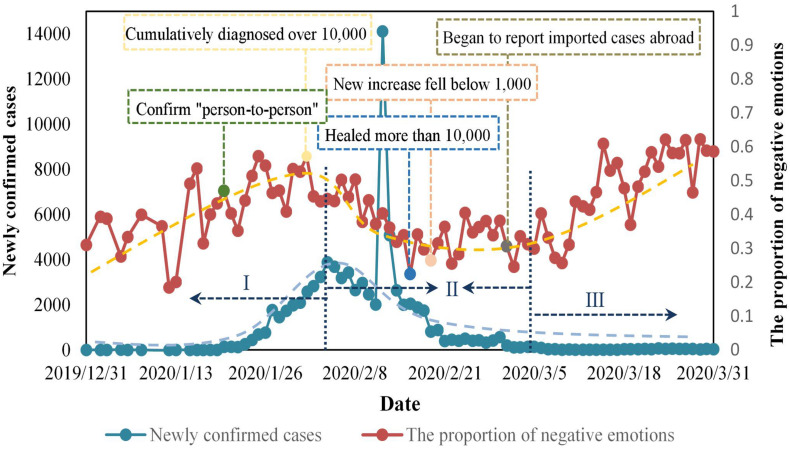
Changes in the proportion of people’s negative emotions and the number of newly confirmed cases.

**FIGURE 6:**
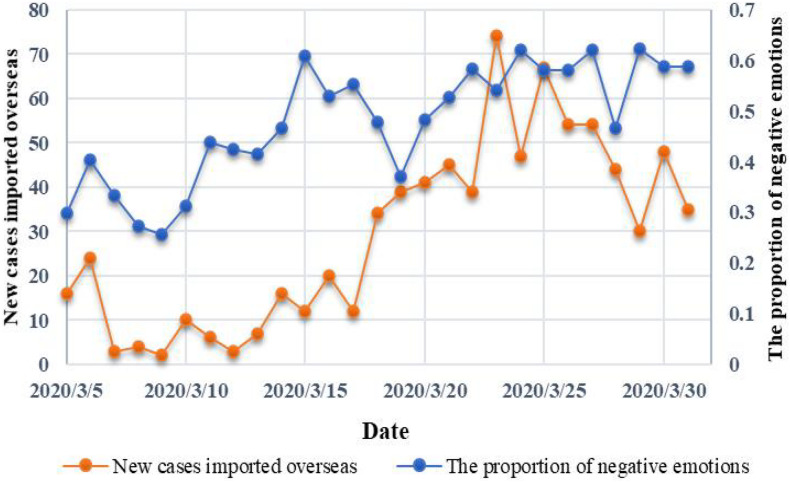
Changes in the proportion of people’s negative emotions and the number of newly imported cases abroad.

Figure 5 depicts the changes of new confirmed cases and the proportion of negative emotions of Weibo users over time. As can be seen from their trend lines, during the growth period and decay period of the epidemic, the proportion of negative emotions of Weibo users has a similar trend with the number of new confirmed cases. The number of newly confirmed cases increased during the growth period and the proportion of people’s negative emotions also increased, while both decreased during the decay period. At some important epidemic nodes, the proportion of people’s negative emotions will also show similar changes. For example, the proportion of people’s negative emotions at the time points of “confirm person to person” and “cumulatively diagnosed over 10,000” was at the peak. Besides, the proportion was at a lower level at the time points of “new increase fell below 1,000” and “healed more than 10,000.” However, in the extinction period of the epidemic, the proportion of people’s negative emotions and the number of newly confirmed cases have different patterns of change, showing an obvious upward trend. Through observation and analysis of the review information, we find that people’s discussion points at this stage have changed from newly confirmed cases to imported cases abroad. Moreover, the increase in the proportion of negative emotions was roughly consistent with the time when People’s Daily began to publish statistics on imported cases from abroad. Therefore, we speculate that the change in people’s emotions at this stage may be related to the number of new cases imported overseas. Figure 6 depicts the trend of them over time, and we can see that there is a high similarity between people’s emotions and the number of new cases imported overseas. We calculated the correlation between some statistical indicators of the epidemic and the proportion of negative sentiments in these three stages, and the results are shown in Table 5, which further validates our above conjecture.

**TABLE 5:** Correlation between epidemic indicators and the proportion of negative sentiments.

**Stage**	**Newly cured**	**New deaths**	**Newly imported**	**Newly confirmed**
Stage I	–0.13	0.02	\	0.67
Stage II	–0.09	–0.06	\	0.55
Stage III	–0.23	–0.13	0.73	–0.23

In summary, we conclude that changes in the epidemic situation will indeed affect the emotions of Weibo users. The proportion of negative emotions of Weibo users showed a similar trend with the number of newly confirmed cases in the growth and decay period of the epidemic, and the same trend with the number of newly imported cases in the extinction period. And a better control of the epidemic would help the public to convey positive emotions.

### Gender Difference

**FIGURE 7:**
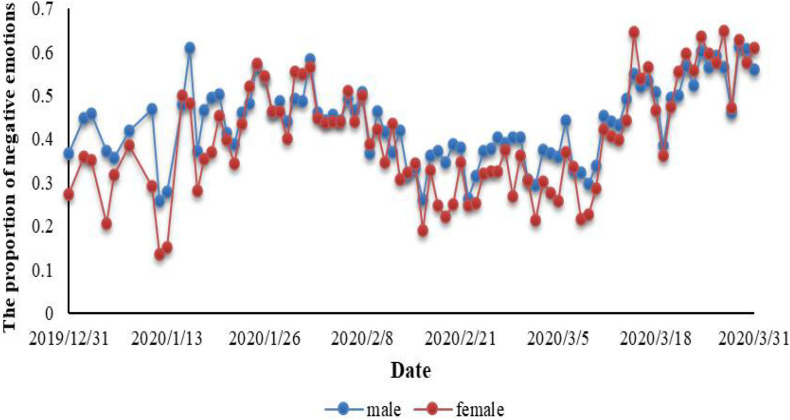
Gender differences in the proportion of negative emotions.

In addition, we have drawn some interesting conclusions. Figure 7 depicts the change in the proportion of male and female negative emotions over time during the epidemic. It can be seen that in the three stages of the epidemic, the proportion of negative emotions in men was generally higher than that in women. We conducted variance analysis on them, results are shown in Table 6. The *P*-value of significance test was 0.03748 < 0.05, effect size (η^2^) = 0.03 > 0.01, indicating that there is a strong significant difference between them, but the difference amplitude is small.

**TABLE 6:** Variance analysis of gender differences.

Group	N	Sum		Mean		Variance	
Male	86	37.94		0.44		0.008	
Female	86	34.90		0.41		0.016	

**Source of difference**	**SS**	**df**	**MS**	**F**	***P*-value**	**F-crit**	**Effect size (η^2^)**

Between groups	0.05	1	0.05	4.40	0.04	3.90	0.03
Within groups	2.08	170	0.01				
Total	2.13	171					

### High Influence Groups and Low Influence Groups

**FIGURE 8:**
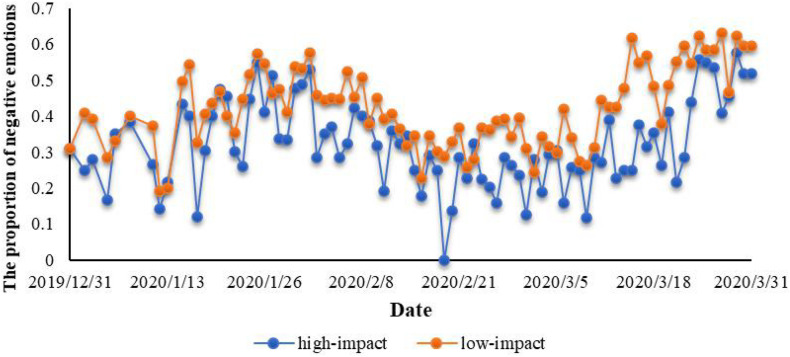
Difference in the proportion of negative emotions between high-impact and low-impact groups.

Another interesting conclusion is that high-influence people show more positive emotions than low-influence people. We divide the users with ≥ 10,000 fans in the data set into a high-impact group. On the contrary, users with less than 10,000 fans are classified as low-impact groups. The reason for this segmentation is that we found that the number of fans = 10,000 is a mutation point through the data distribution, which has the most obvious difference after grouping. Figure 8 depicts the change in their negative emotional proportion over time. It can be seen that the proportion of negative emotions of people with low influence is significantly higher than that of people with high influence. In the same way, ANOVA was conducted on them, the results are shown in Table 7. The test statistic *P* = 1.71E-08 < 0.01, effect size (η^2^) = 0.17 > 0.14, indicating that the difference between them has strong significance and magnitude.

**TABLE 7:** Variance analysis of differences between high-impact and low-impact groups.

Group	N	Sum		Mean		Variance	
High-impact	86	27.80		0.32		0.014	
Low-impact	86	36.75		0.43		0.012	

**Source of difference**	**SS**	**df**	**MS**	**F**	***P*-value**	**F-crit**	**Effect size (η^2^)**

Between groups	0.47	1	0.47	35.08	1.71E-08	3.90	0.17
Within groups	2.25	170	0.01				
Total	2.72	171					

## Discussion

COVID-19 is the most serious public health incident in recent years. Standardizing and guiding public emotions and ensuring people’s epidemic prevention and control is a core task. During the epidemic in China, social media such as WeChat and Sina Weibo played a vital role in disseminating government information and public activities (Xu W. et al., 2020). This paper analyzes the comments on Weibo to explore the emotional changes of the Chinese people during the epidemic.

First of all, from the perspective of word frequency statistics, government work is the topic of most concern to the public. The fear of illness, the “blocked” situation, the high degree of uncertainty about the future, and the sense of financial insecurity exacerbate the stress, anxiety, and depression experienced by people (Király et al., 2020). In addition, the research results of Dong et al. (2020) prove that public sentiment is related to rumors spread on the Internet. Therefore, the government should reassure the people in a timely manner and maintain the security of online public opinion.

Second, our research found that there is a strong correlation between changes in the epidemic and changes in people’s emotions. Chen et al. also constructed the relationship model between the dominant public opinion and entity behavior in each stage of epidemic situation by using the frequency and probability of keywords in the emotion category (Chen et al., 2020). These findings may help to predict the emotional swings people are likely to experience in advance, and to develop effective coping strategies. In addition, according to the emotional characteristics of different groups, the government can better transmit positive energy. For example, use the positive attitude and social attention of high-influence groups to guide people to relieve anxiety and psychological pressure.

Our research also has some limitations. First of all, this paper only divides emotions into positive and negative categories without further subdivision, and cannot identify the specific emotions expressed by user comments, such as happiness, sadness, anxiety, fear, etc. Secondly, we just found the potential regularity between the trend of epidemic change and emotional change, and did not conduct in-depth quantitative research on how the development of the epidemic affects people’s emotions. Finally, the changes of public opinion reflected by a single social network platform may lack some representativeness. In the future, we plan to conduct more in-depth research to explore the more specific and close internal relationship between the epidemic and emotional changes.

## Data Availability Statement

The original contributions presented in the study are included in the article/Supplementary material, further inquiries can be directed to the corresponding author/s.

## Ethics Statement

Ethical review and approval was not required for the study on human participants in accordance with the local legislation and institutional requirements. Written informed consent for participation was not required for this study in accordance with the national legislation and the institutional requirements.

## Author Contributions

WP and S-jL contributed to study design. W-qD, GH, R-jW, W-lP, and CH collected and analyzed the data. W-qD interpreted results. WP, W-qD, GH, and S-jL wrote the manuscript. All authors revision of the manuscript and the final approval of the version to be published.

## Conflict of Interest

The authors declare that the research was conducted in the absence of any commercial or financial relationships that could be construed as a potential conflict of interest.

## Publisher’s Note

All claims expressed in this article are solely those of the authors and do not necessarily represent those of their affiliated organizations, or those of the publisher, the editors and the reviewers. Any product that may be evaluated in this article, or claim that may be made by its manufacturer, is not guaranteed or endorsed by the publisher.
